# Context and Complexity in Telemedicine Evaluation: Work Domain Analysis in a Surgical Setting

**DOI:** 10.2196/26580

**Published:** 2021-09-16

**Authors:** Hedvig Aminoff, Sebastiaan Meijer

**Affiliations:** 1 Division of Health Informatics and Logistics Department of Biomedical Engineering and Health Systems KTH Royal Institute of Technology Stockholm Sweden

**Keywords:** telemedicine, telemedicine evaluation, ERCP, work domain analysis, abstraction hierarchy, complexity, context, cognitive systems engineering

## Abstract

Many promising telemedicine innovations fail to be accepted and used over time, and there are longstanding questions about how to best evaluate telemedicine services and other health information technologies. In response to these challenges, there is a growing interest in how to take the sociotechnical complexity of health care into account during design, implementation, and evaluation. This paper discusses the methodological implications of this complexity and how the sociotechnical context holds the key to understanding the effects and outcomes of telemedicine. Examples from a work domain analysis of a surgical setting, where a telemedicine service for remote surgical consultation was to be introduced, are used to show how abstracted functional modeling can provide a structured and rigorous means to analyze and represent the implementation context in complex health care settings.

## Introduction

### Overview

Time has shown that it is difficult to scale up successful telemedicine innovations, and telemedicine has long been fraught with critical dilemmas regarding implementation, adoption, and evaluation [1-4]. In order to move forward from the repeated and seemingly paradoxical failures of telemedicine [2] and health information technologies, there have been calls for research and design methodologies that can address the many levels of complexity in health and care [5,6]. In this paper, we present reasoning for why it is important to apply a “complexity lens” to understand baseline conditions prior to technology implementation and evaluation in health care settings, and a rationale for why mapping the context provides important keys to understanding clinical outcomes and adoption when new health technology interventions are introduced. We describe how principles from complexity science can be applied in a structured and rigorous analysis of a telemedicine implementation context through work domain analysis [7-9]. Work domain analysis is a type of modeling specifically developed to design and analyze complex, adaptive sociotechnical systems. We include examples of how the method was used to analyze and represent many different sources of complexity that shape work in a surgical setting [10].

### Context and Complexity in Health Technology Implementation and Evaluation

It is generally acknowledged that health technology implementation and outcomes are affected by contextual factors, and it is extremely difficult to scale up demonstration projects [5]. Despite this, few studies account for the preconditions for implementation in a way that adequately captures the inherent complexity of health care or in a fashion that can inform systems development or assessment. Technological interventions in health care are generally complex, as are the health care settings in which they are used, and there is a demand for a methodological shift when studying the introduction of new technologies in this type of context [11-13].

**Figure 1:**
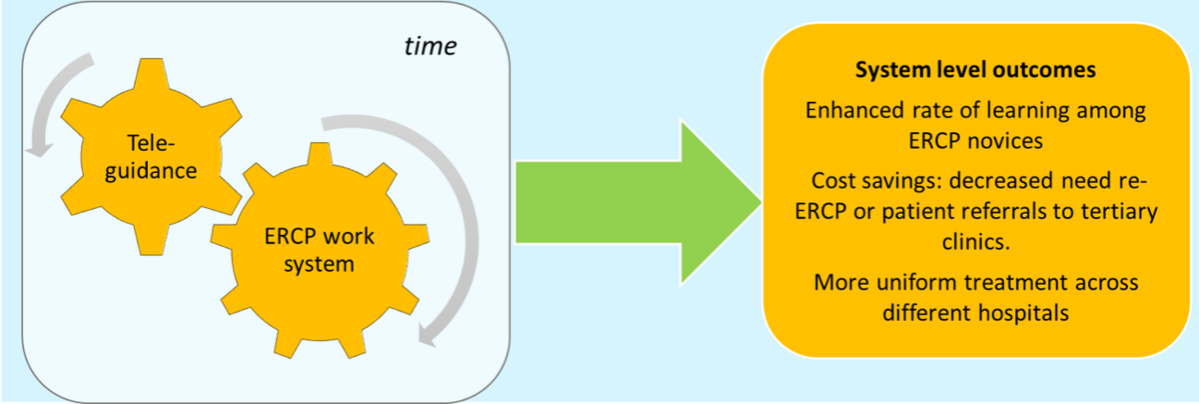
System level outcomes. ERCP: endoscopic retrograde cholangio-pancreatografy.

We encountered a number of challenges when we attempted to provide a baseline description of the implementation context when a telemedicine system for remote surgical consultation was to be scaled up to multiple hospitals. If the service was adopted and used over time, it was expected to improve clinical outcomes and provide educational benefits. However, there were differences between the hospitals (eg, in work practices and resources), which potentially could interact with adoption and even lead to abandonment [5]. In addition to the inherent complexity of the clinical procedure and patients’ conditions, introducing new technology for surgical collaboration introduced new sources of complexity; for instance, the telemedicine system would bridge several technical systems and social and professional workgroups at different hospitals. These factors together would contribute to system-level outcomes over time (Figure 1).

It was unclear to us how technical, social, and organizational factors set the implementation sites apart, how to conduct the analysis, and how to represent the findings in a way that could be useful for stakeholders.

A systems engineering method called work domain analysis (WDA) [7-9] appeared to be a suitable method for our purposes. WDA is intended to analyze complex work systems, and there are examples where it has been used in health care [14-19]. However, in our case, we wanted to capture and contrast domain-specific contextual factors on multiple work system levels at multiple sites, and this broad, explorative scope of analysis presented a particular modeling challenge. We eventually found a solution to this dilemma through iterative modeling, whereby we found a way to construct multiple, complementing models of the domain.

In the following sections, we provide a background for our choice of method.

### Evaluating Telemedicine

There are many reasons for evaluating new technologies in health care and many ways to do this. While it might seem evident that new telemedicine systems should be evaluated for clinical, policy, and economic reasons, this is not always the case. In 2010, only 20% of the WHO (World Health Organization) members reported having published an evaluation or review on the use of telemedicine in 2006 [20]. A review of evaluations studying deployed hospital-to-hospital telemedicine services up to May 2016 only identified 164 papers [21].

Telemedicine evaluation raises many research questions [22], and different stakeholders have different expectations about what an evaluation should provide: evaluations can include organizational, technical, social, ethical, and legal, as well as transferability aspects [23]. Among peer-reviewed clinical evaluations of telemedicine interventions in hospital facilities, half were evaluated in terms of clinical outcomes and economic or satisfaction measures, while the other half were descriptive reports with ad hoc structure [21].

### Telemedicine as an Intervention

In clinically-oriented research, telemedicine can be described as an intervention, which emphasizes its function in a clinical process [24]. Evaluating an intervention’s effectiveness and efficacy is central to health care quality since practice recommendations should be grounded in high-quality evidence, and policy and funding decisions should also have a sufficient basis [25]. Randomized controlled trials are a gold standard for ascertaining the efficacy of pharmaceuticals or clinical treatments with well-defined active components. This type of study is designed to show the size of a clinically meaningful benefit and the likelihood that this result is caused by the intervention (ie, that the intervention causes an effect X with size Y, with a confidence interval of Z). In addition, the results can show that an intervention is safe and effective [26].

Controlled studies are feasible for interventions with a limited number of readily defined components and a known mechanism. However, in the case of complex interventions [27] such as telemedicine [28], the “active components” can be difficult to define, and it might not be entirely clear what changes can be expected or how change will be achieved [29].

In response to the challenges of evaluating complex interventions, there is guidance recommending that process evaluations be conducted alongside trials of complex interventions to help provide the information required to interpret the findings of a specific evaluation and to generalize findings to other contexts [29]. Three major themes in process evaluation are implementation, mechanisms, and context [27].

### Implementation, Mechanisms, and Context

Implementation research has identified and compiled a multitude of contextual factors that can influence how new treatments and new ways of working are accepted and adopted [30,31]. The profusion of contextual factors that can affect an intervention reflects the reality of health care settings, where there is generally very much going on. Guidance for evaluating complex interventions also recommends that the intervention and its assumed causal mechanisms be clearly described, but this is difficult to achieve without a systematic description of the implementation context [32].

While context is considered to be a major concern for outcomes of complex interventions, the term remains “a slippery notion” [33], which is inconsistently defined and conceptualized [30,31,34]. In complex, adaptive work systems, which per definition are intractable, it is untenable to identify and measure all potential determinants [35]. The “variables paradigm” [36] provides output such as lists and categorizations of key variables and evaluation elements, yet is unlikely to provide means to identify which possible factors are most likely to influence outcomes in a particular case or how these factors interact once an intervention is introduced [33].

In-depth case research using sociological and organizational research methodologies contrasts to predominant “mechanistic” conceptions about implementation and component-oriented research [37] and can provide keys to the mechanisms of implementation and adoption through detailed experiential and contextual information [38,39]. The internal validity of such approaches is gained by the authenticity of observations and interpretations, which can cause weaknesses (eg, in quality case reports where a few members recount their experience of an intervention and its impact) [40]. In both case study research and quality reports, results are often presented in the form of detailed accounts, interspersed with quotes from participants [40]. This format can capture the uniqueness of a case but simultaneously raises questions about what has been accounted for, how lessons can be transferred to other interventions, or how the same intervention may have played out in another setting.

Common trial reporting formats and health technology assessments generally demand limited information about an intervention’s implementation or context [41]. Yet without this information, generalizing findings beyond a specific case becomes difficult. It also becomes challenging to determine whether changes detected during a study are due to the intervention or if its implementation or context is causing the effects [26].

These insights have generated calls to apply concepts from complexity theory during implementation and evaluation [6,13,29,42].

### Complexity in Health Care

In health services research, “complexity science“ has been used as an umbrella term, referring to the use of concepts about complex adaptive systems as a response to the increasing complexity and rapid rate of change and of health care [43,44]. “Complexity science” may invoke associations to definitions and methods used to address computational complexity and natural systems, and its use in health services research has invoked some criticism from proponents of “hard” approaches to complexity [45]. However, complexity science ideas have been, for example, used to inform theories and frameworks for evidence translation, implementation, and evaluation [30,39,46,47].

Thus far, health services research has mainly used complexity concepts to “sensitize” and support evaluation and also help identify issues that need to be managed during implementation, for example, in workshops to identify or solve specific problems, increase collaborative practices, or identify barriers to change [48]. However, complexity concepts have been inconsistently applied [48] or merely used as an abstract explanatory tool when they can be disciplined and refined to match specific research questions [49]. Moreover, the superficial use of complexity concepts runs the risk of fixating on easily identified components of a system, or effects from the context, without breaking adaptations apart or systematically taking interactions into account [50,51]. Another effect can be that different levels of work systems are separated and studied by different disciplines [52] or approached through different studies [47].

However, there is an option to apply systems engineering and research methodologies developed specifically for complex settings. Within the field of human factors or ergonomics, there is a long history of employing systems approaches in research and design of human-technology interactions [53], with concepts and definitions that explicitly address behavioral and organizational factors. The field provides theory and engineering methodologies employed in regulated, high-performance, and safety-critical domains such as aviation, the nuclear industry, and defense, but which are also well-suited for health care [54-56]. These types of work systems share characteristics; work is conducted by humans and technology, with operators balancing performance, quality, and safety with the demands set by uncertainty and rapid technological and organizational change.

### Complex, Adaptive Sociotechnical Systems

Health care settings such as hospitals can be defined as complex, adaptive sociotechnical systems [57,58], where interactions among technical, human, and organizational elements generate complexity in many dimensions. This implies that efforts to induce system change through new technology can be expected to affect patterns of interaction within the system and between the system and its context [59,60]. These patterns of interactions make it challenging to scale up innovations from one context to another [61] and also make evaluation difficult, as it is hard to link technological change to specific outcomes [62].

One value of a sociotechnical systems approach lies in acknowledging the variable and irregular nature of health care work, where staff continually monitor and adapt to changing circumstances to uphold safety and performance while helping patients and balancing organizational demands, social and professional values, and norms [63]. This intentional, adaptive behavior generates the system’s “self-organizing” capacity, making these work systems resilient [64] and providing keys to understanding and describing the system.

Principles from theory about complex adaptive systems emphasize the need to understand initial conditions as a baseline, and a “map” of the context and the rules governing behavior are important for understanding system behavior [6,65].

### Cognitive Systems Engineering

Cognitive systems engineering (CSE) is a field of research and design in complex, adaptive sociotechnical systems [66-68]. CSE research focuses on how designed artifacts interact with their environment and with the humans using it. CSE practice includes eliciting and defining requirements and designing and evaluating human-technology work systems [69].

Cognitive work analysis (CWA) [8,70] is a formative CSE framework that provides functional analysis methods for design and evaluation. CWA is rooted in traditions and concepts from ecological psychology [71], distributed cognition [72], and expert decision-making in naturalistic settings [73], which emphasize how mutual interactions between the environment and agents shape behavior.

CWA consists of five phases, the first of which is called WDA [8,9]. WDAs are typically performed to provide a shared representation of complex work systems in the face of technological change ahead of system development or acquisition (eg, during the design requirements and specifications phases) [9]. WDA focuses on modeling the contextual factors which shape actors’ behaviors. The idea is to create a complete picture of the workers' problem space by specifying what is to be achieved and the values, priorities, functions, and physical resources that affect this work.

This is done in abstracted, functional terms rather than through details of objects or tasks. Representing the sociotechnical context in this abstracted format can support the exploration of how the affordances of physical objects interact with functions towards system goals and how expanding the system with new components may impact the system as a whole [74].

### Can Work Domain Analysis be Applied in Health Care Work Systems?

CWA originated in analyses of well-defined, tightly coupled causal systems (ie, engineered systems that are constrained by natural laws and technical factors) [7]. Some have claimed that WDA is not well-suited for health care [75], where system behavior is characterized by intentional constraints, such as actors’ goals, values, priorities, and shared rules of practice.

Health care systems are generally open systems, with many external interactions. This means that it is difficult to define system boundaries and distinguish discrete components and mechanisms that are “internal” to the system and how they interact with the “outer environment” [76]. As a consequence, boundaries between what is internal and what is external will be conceptual, an artifact, rather than ontological [8], and must be decided with careful consideration of the purpose of the analysis [9].

However, WDA has been used to model numerous intentional, open systems(eg, in naval command and control, ambulance dispatch, and health care) [77-79]. Jenkins et al suggest that if suitably adapted, WDA models can be utilized to predict and evaluate system-level outcomes when new technologies are introduced in sociotechnical systems [62].

### Understanding the Implementation Context for Teleguidance in ERCP

The telemedicine service we studied was developed to enable real-time, professional-to-professional video collaboration during endoscopic retrograde cholangio-pancreatografy (ERCP), a technically advanced endoscopic procedure for biliary and pancreatic disease. The telemedicine service, which came to be called teleguidance, had demonstrated clinical and economic benefits in a feasibility study [80]. Health-economic modeling also showed the potential for positive clinical and economic outcomes [81]. This provided a rationale for scaling up the practice and an interest to generate additional evidence for the new way of working.

Quantitative clinical data was to be collected to investigate the clinical effectiveness of teleguidance. However, the service also had to be used over time for any desired quality improvement outcomes to come into effect. So there was also interest to conduct a qualitative inquiry to understand whether conditions at the various sites might influence any clinical results or affect how teleguidance would be adopted and assimilated into everyday practice (Multimedia Appendix 1).

The complexity of the highly specialized surgical procedure and the hospital settings made it neither feasible nor theoretically justified to choose an ad hoc number of components (eg, from a determinant framework) [31], and also expect to achieve an adequate understanding of how the implementation context could influence the use and outcomes of the new technology [11]. Without a method adapted for the complexity of the work systems involved, our attempt at understanding and describing the implementation context might also fail to account for interactions between different parts of the system, or from the context, in a systematic way.

We observed WDA was seen as a candidate method as it is developed to accommodate sociotechnical complexity and would provide a structured and accountable analysis. Representing constraints that shape system behavior in an abstracted manner would be useful for comparing different sites and could also support the prediction of change and unintended consequences, which is a central aspect of complexity-informed evaluation [6].

## Methods

### Data Collection and Analysis

We used an ethnographical approach with extensive fieldwork and interviews to collect data and generate a deep understanding of the context in a working system [82]. This included three iterations of data collection using a sequence of techniques, moving from a general “rough” level of description and understanding to a finer grain.

Observations and interviews were conducted at the central and remote sites. A total of 20 semistructured interviews with 10 ERCP specialists, 5 ERCP assistants, 3 technical staff, and 2 administrative staff from 5 hospitals were conducted. During the data collection phase, a service blueprint [83] was designed, which served as an intermediary, shared representation [84] (Multimedia Appendix 2). Interviews were transcribed, and thematic coding [85] was conducted, using predetermined categories and prompts (Multimedia Appendix 3) pertaining to the abstraction levels described by Naikar [9] from the WDA framework.

Data collection and analysis are described in more detail in an adjacent paper, “Modeling implementation context in telemedicine” [10].

### Defining System Boundaries

We wanted to conduct a broad and deep investigation of the ERCP work context at the participating sites. Our broad interpretation of the implementation context was based on a view that implementation and use of teleguidance will be shaped by physical and organizational constraints, which are situated within the functional goals and values of the work domain [86].

When analyzing a system, it is generally deemed necessary to define system boundaries in order to distinguish components and mechanisms that are “internal” to the system, how they interact with the “outer environment,” and their functional relationships [78]. However, work system complexity became apparent early on during observations and interviews, and it became clear that it would be difficult to set clearly defined system boundaries.

During procedures, the ERCP team works within a specific physical space to perform a specific type of procedure during a limited time frame. However, before and during each ERCP procedure, there were continual trade-offs between clinical work and organizational demands (eg, demands for resource efficiency), which also sometimes conflict with clinical priorities. It also became clear that administrative and clinical roles and tasks were highly interwoven in ways that were not necessarily reflected by formal roles or organizational boundaries. Similarly, development work, such as research and training, was continually ongoing, and these aspects of work were shaped by other sets of constraints (eg, funding and practice standards, which were controlled by sources other than the hospital administration).

In addition, the clinical work system at the university hospital underwent substantial reorganization during the study. Similar but less extensive reorganizations were taking place at several of the smaller hospitals.

Hollnagel reframes the question of system boundaries and context by speaking in terms of foreground and background functions, rather than strict system boundaries [87]; as such, functions and constraints which are central during ERCP procedures could be included in the WDA model without explicit reference to whether they lay within an arbitrary system definition or not.

### Creating the Models

The abstraction hierarchy was constructed using Naikar [9] as a main resource and iteratively modeling our findings and revisiting the purpose of the analysis, with feedback from clinical practitioners and project managers.

The first iteration of the abstraction hierarchy centered on structuring the large amounts of data collected. Findings from interviews, observations, and documents were entered into a large general-purpose spreadsheet. The findings were categorized into abstraction levels and linked to cells in the matrix.

Multimedia Appendix 4 shows the sequence in which we populated different levels of the abstraction hierarchy matrix and provides examples of the questions used to guide the work.

The second iteration of the abstraction hierarchy mainly focused on testing different ways of ranging and decomposing the clinical work system to make the analysis tractable. The interactions between clinical work and organizational demands made it challenging to define a part-whole systems decomposition of the hospitals and their subsystems according to organizational boundaries. We decided to represent “hospital” as the overarching system, with a partial abstraction hierarchy, and “ERCP work” as a conceptual subsystem, with a detailed abstraction hierarchy. The ERCP work system is loosely bounded [78], meaning ERCP practitioners have control over some resources but not of the whole system. Capturing this aspect was a conundrum until we decided to create multiple models of the domain. Naikar [88] proposes representing certain domains as having distinct facets. This is considered a way to handle the wide range of constraints necessary for this type of intentional system.

Therefore, a decision was made to construct multiple models of the domain, one representing the “primary” clinical work and the other representing the “secondary” functions that provide the infrastructure and resources for the clinical work, such as administration and management and training and research. The three facets represent aspects of the same clinical work system, yet each facet is seen as separate through the nature of tasks and aspects, such as organizational departments, competencies, and roles. Individual stakeholders can be involved with more than one of the three facets, as is the case with senior doctors and nurses who have managerial roles in addition to their clinical functions.

We considered development work (research, education, and training) to be distinct from other secondary functions, and we finally represented the domain as three facets: treatment, development, and administration.

The third iteration was an exercise in improving the internal structure of the means-ends relationships within the conceptual framework of the functional facets. The constraints were decomposed in detail on certain levels of abstraction but are aggregated in the presented model for increased legibility.

### Using the Models to Proactively Identify Implementation Issues and Possible Outcomes

By tracing the many-to-many means-ends relationships among constraints through “how-what-why” reasoning [74], the abstraction hierarchies served as a simple artifact to investigate possible scenarios when teleguidance is used (Multimedia Appendix 5)

## Results

**Figure 2:**
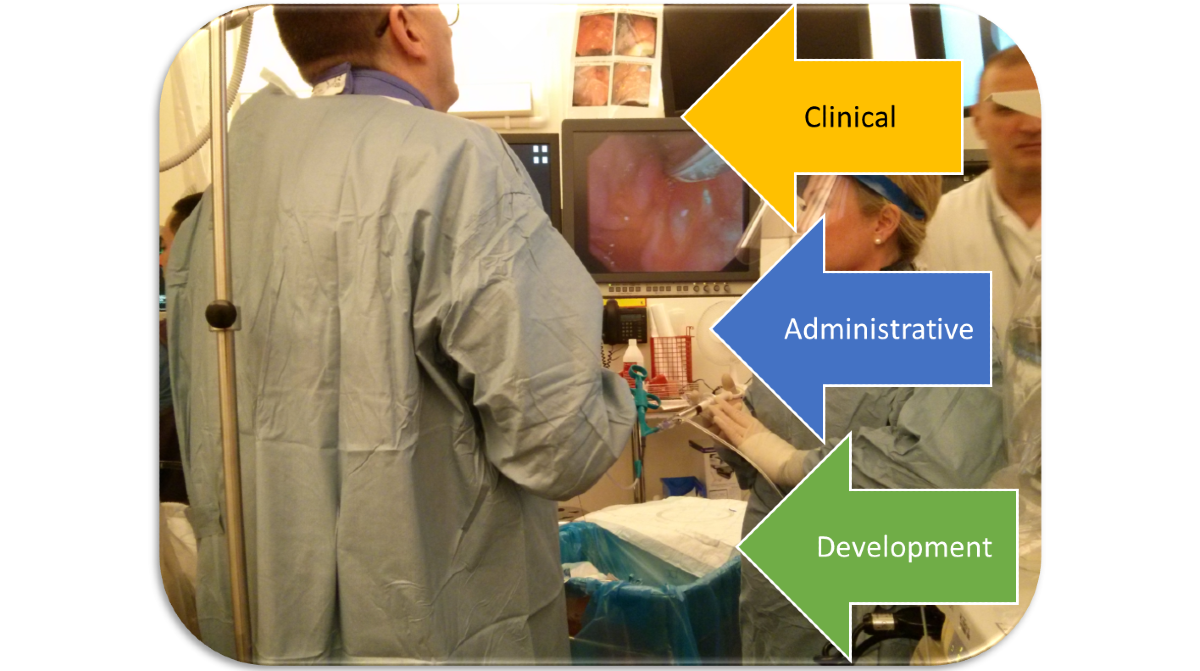
Three functional facets of the endoscopic retrograde cholangio-pancreatografy (ERCP) work domain.

Three functional facets of the domain were modeled (Figure 2) and are defined as follows:

- Clinical: The treatment of patients through ERCP (Multimedia Appendix 6).
- Development: Functions such as developing clinical methods and technology; research (eg, developing clinical methods and tracking outcomes), collaboration with suppliers, and arranging and providing supervision and training opportunities (Multimedia Appendix 7).
- Administration: Support functions for the clinical work, such as managing finances and staff and facilities, including IT (information technology) and medical technology (Multimedia Appendix 8).

**Figure 3:**
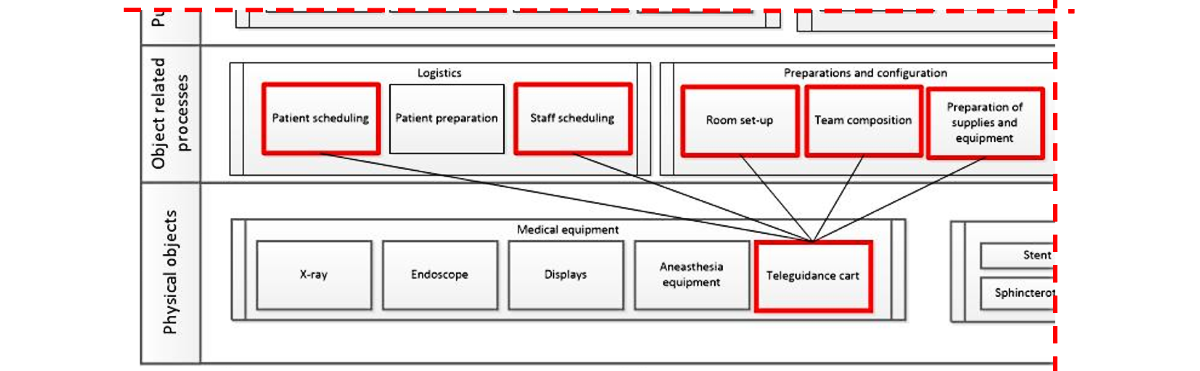
Cropped image of an abstraction hierarchy showing how the model can be used to trace and visualize interactions, in this case between physical objects and work processes.

The models helped us structure findings from the interviews, and we could link the physical teleguidance equipment to reverberations in logistic processes, such as patient and staff scheduling and preparations for ERCP procedures (eg, set-up, preparation of supplies, and team composition; Figure 3).

The abstraction hierarchies enabled us to explore possible scenarios during and after implementation. For example, we could identify issues that might be of importance during implementation and be weighed in during evaluation, such as if teleguidance affects the duration of procedures or the time to prepare for procedures.

A detailed account of specific findings is provided in “Modeling implementation context in telemedicine” [10].

## Discussion

### Overview

Despite general agreement that context is important and that “complexity science” can be of value, it appears that in practice, it is difficult to describe and analyze contextual factors and, at the same time, accommodate complexity during evaluation. Furthermore, context is often given a minor role in studies of health technology implementation or health technology assessment [30]. We decided to attempt a broad analysis of the implementation context for a telemedicine service by conducting a WDA. However, it was initially unclear if the scope and open, intentional nature of the work systems would be a problem.

### Principal Findings

**Figure 4:**
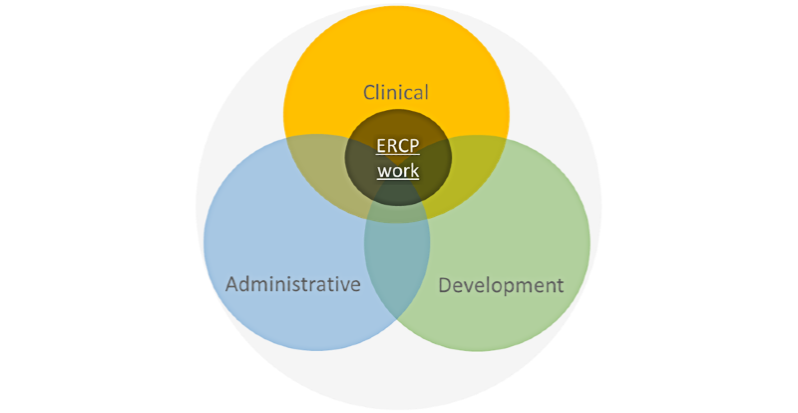
Three functional facets of the domain which shape ERCP work. ERCP: endoscopic retrograde cholangio-pancreatografy.

In order to represent the entire problem space that clinical practitioners face during ERCP, we discovered that it was relevant to include aspects of nonclinical work to the extent that they affected clinical procedures. To handle the width and depth of this scope, we conceptualized and modeled three functional “facets” of the domain which shape ERCP work (Figure 4).

Each facet represents a set of constraints that shape ERCP team members’ work before, during, and after each ERCP procedure. With Hollnagel’s terminology [87], the functions and constraints within the administrative and development facets can loosely be considered as background to the functions and constraints in the clinical facet.

The models helped us explore the dynamics of the work systems and project possible interactions during the use of the telemedicine service. An example of findings was that despite shared clinical goals across the collaborating sites, relevant aspects of the administrative and development facets need similar coordination across hospitals. While this may seem obvious, these factors are beyond the control of the clinical staff and may interfere with teleguidance use over time. The WDA helped us identify and represent these types of issues in detail [10].

### Defining System Boundaries

Initially, we set wide system boundaries to include many explanatory variables. During the data collection phase, it became increasingly difficult to establish the boundaries for the work system and decide what should be included in the WDA. A narrow definition of the unit of analysis, for example, ranging the system according to what goes on within the physical space of the operation theater during ERCP, would have given a precise ontological boundary. However, this could have excluded organizational factors that have a bearing on ERCP performance and would therefore forfeit the purpose of the analysis, which was to map constraints that could come to interact with the implementation of teleguidance.

### Practical Aspects of Conducting a WDA

The design, implementation, and assessment of the telemedicine service we evaluated was a transdisciplinary effort involving clinical practitioners and researchers, alongside human-computer interaction and project management experts. Consequently, there were different expectations about what an evaluation should provide, and the practical application of WDA requires an understanding of systems-theoretical concepts. While Rasmussen [7,70] and Vicente [8] provide comprehensive but somewhat conflicting descriptions of how to conduct WDA, Naikar provides a systematic methodology [9]. However, it was challenging at first to construct an abstraction hierarchy due to the width of the analysis. We also had difficulties establishing a hierarchical decomposition of the work systems, as work in practice did not necessarily follow organizational boundaries.

Technical and physical factors are generally more straightforward to distinguish than properties emerging from human intentions. The qualitative methods we used for our deepening investigation required relationships between researchers and domain practitioners, which may be a hurdle due to interprofessional dynamics and hierarchies in health care and time constraints [89].

However, we conclude that the methodology is resource-efficient, especially if the analysis can be reused across multiple problems [90]. The structured, abstracted format is very compact and relatively easy for stakeholders from different disciplines, such as clinical staff and project management, to understand. WDA may be more useful in systems development and evaluations than the narrative accounts common in many qualitative case studies and thereby also be an effective artifact for supporting the interdisciplinary collaboration required for successful human-systems integration [84].

### Conclusion

WDA is a systems engineering method that allowed us to create representations that served as objective models of the implementation context by focusing on functions and constraints shaping work-system behavior. Creating models helped us avoid the notion that context is a fixed entity or can be described by compiling variables or events. The three sets of constraints or facets, which were present in each hospital, represent constraints that shape everyday ERCP work and that can shape the use of teleguidance.

Using abstracted functional modeling guided by theory strengthens the transferability of findings, and the facets can be expected to reflect fields of interest and functions that can affect other telemedicine interventions in similar hospital settings. The structure of the method also supported an iterative “discovery and modeling” approach [91], which was necessary as our understanding of the work systems developed.

We conclude that WDA is an effective method for modeling the implementation context, and that this type of modeling is a practical approach to applying “complexity science” principles, and that it is a way to provide structured analysis without reducing complexity or detailed qualitative accounts common in sociological and organizational research methodologies. The models account for technological, social, and organizational factors and their dynamic interactions, which provide useful information both for policymakers and scientists. The method can be useful for supporting detailed analysis and planning prior to implementation and evaluation of telemedicine, which is currently rare [92].

### Future Work

The three functional facets of the domain (clinical, development, and administration) represent sets of generic constraints that we believe are likely to be present in other hospital environments and likely to affect other technology implementation projects. These can serve as “dimensions” along which to model and analyze similar clinical work systems.

## Appendix

Multimedia Appendix 1 Aims of the clinical study that we were to complement with understanding of the implementation context.

Multimedia Appendix 2 Service blueprint.

Multimedia Appendix 3 Coding categories and criteria for the qualitative data analysis.

Multimedia Appendix 4 The sequence in which we populated different levels of the abstraction hierarchy matrix,.

Multimedia Appendix 5 The abstraction hierarchies served as a simple artifact to investigate possible scenarios when teleguidance is used.

Multimedia Appendix 6 The treatment facet.

Multimedia Appendix 7 The development facet.

Multimedia Appendix 8 The administrative facet.

## Abbreviations

CSE: cognitive systems engineering
CWA: cognitive work analysis
ERCP: endoscopic retrograde cholangio-pancreatografy
WDA: work domain analysis
