# Supplementary figures and images for: Context and Complexity in Telemedicine Evaluation: Work Domain Analysis in a Surgical Setting
Source: JMIR Perioper Med. 2021 Sep 16;4(2):e26580. doi: 10.2196/26580 (PMC8485199; doi:10.2196/26580)

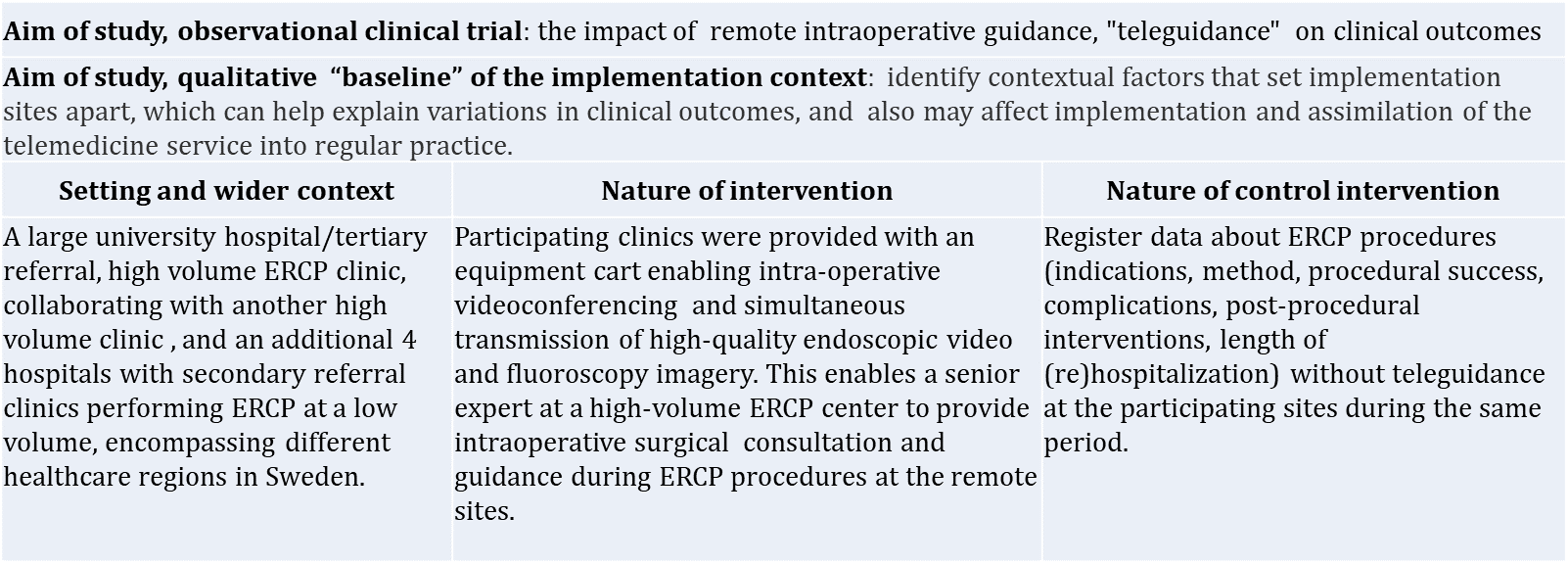

Supplement: Multimedia Appendix 1 [file periop_v4i2e26580_app1.png]

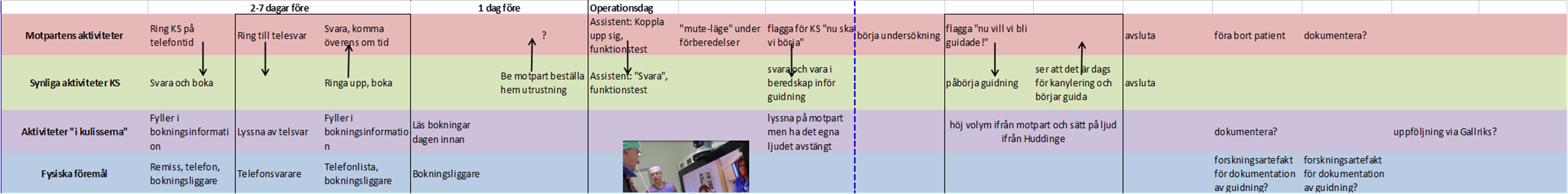

Supplement: Multimedia Appendix 2 [file periop_v4i2e26580_app2.png]

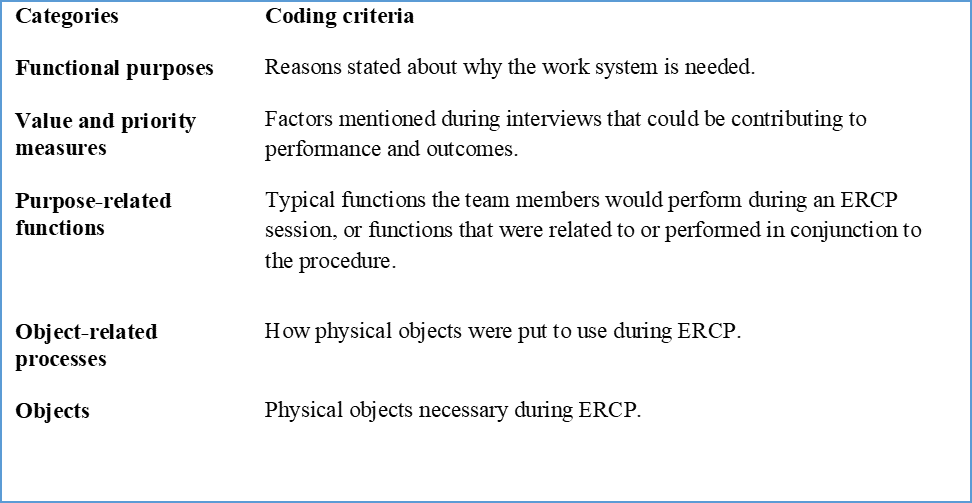

Supplement: Multimedia Appendix 3 [file periop_v4i2e26580_app3.png]

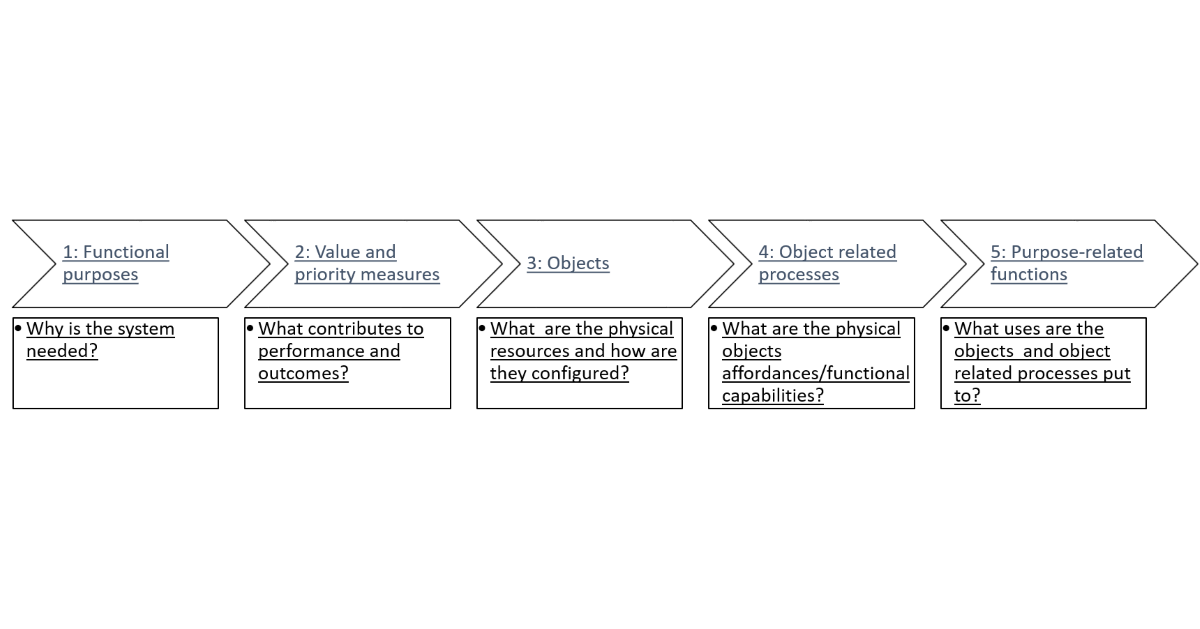

Supplement: Multimedia Appendix 4 [file periop_v4i2e26580_app4.png]

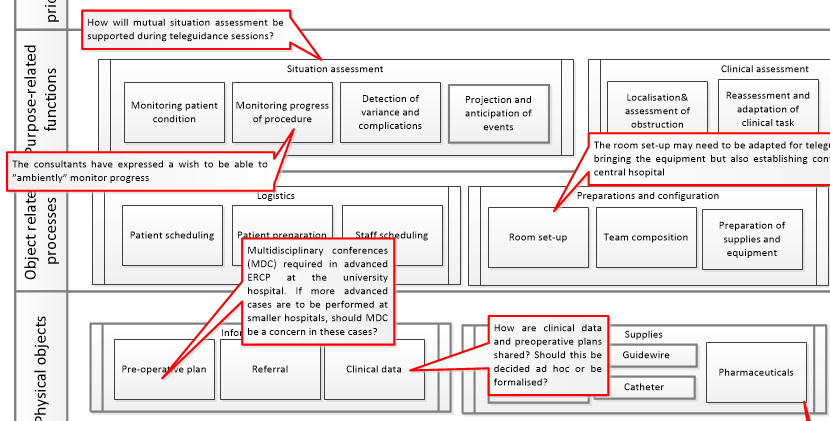

Supplement: Multimedia Appendix 5 [file periop_v4i2e26580_app5.png]

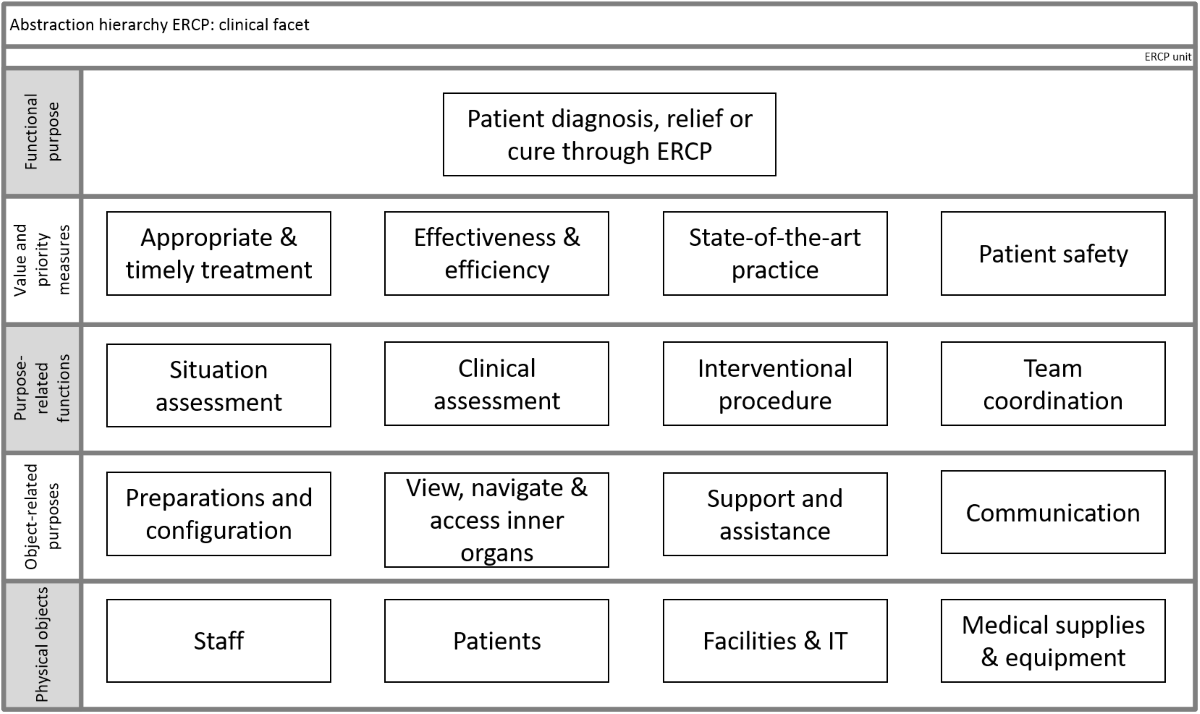

Supplement: Multimedia Appendix 6 [file periop_v4i2e26580_app6.png]

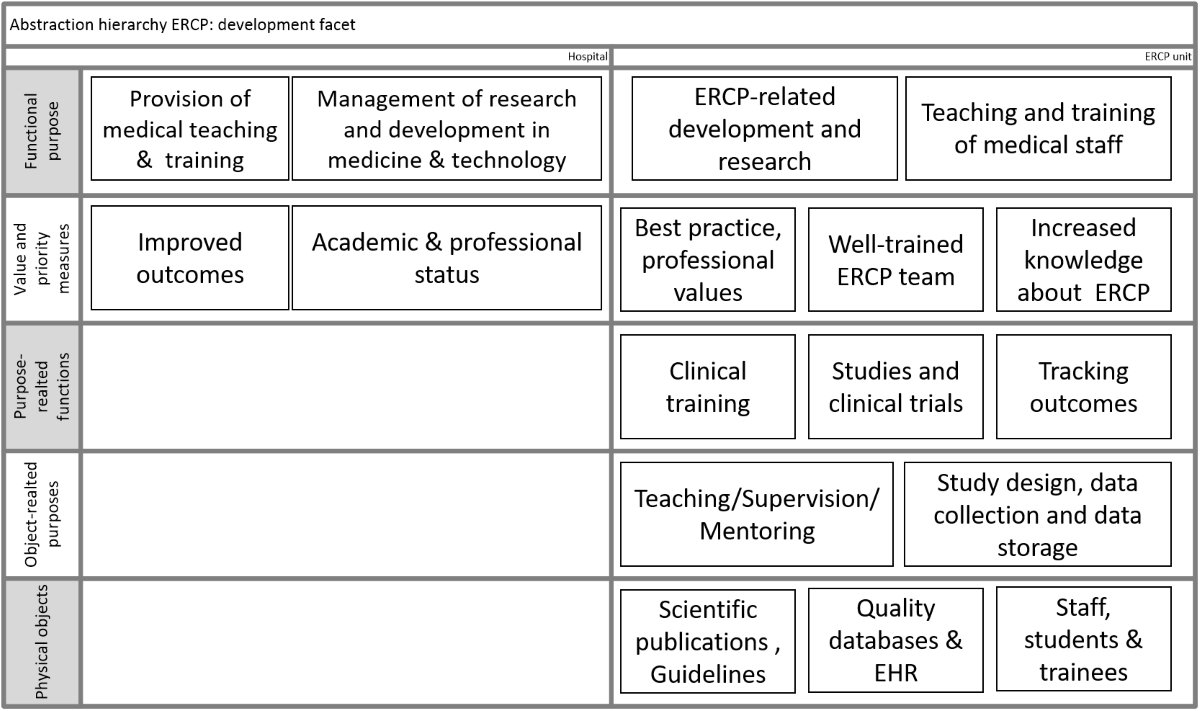

Supplement: Multimedia Appendix 7 [file periop_v4i2e26580_app7.png]

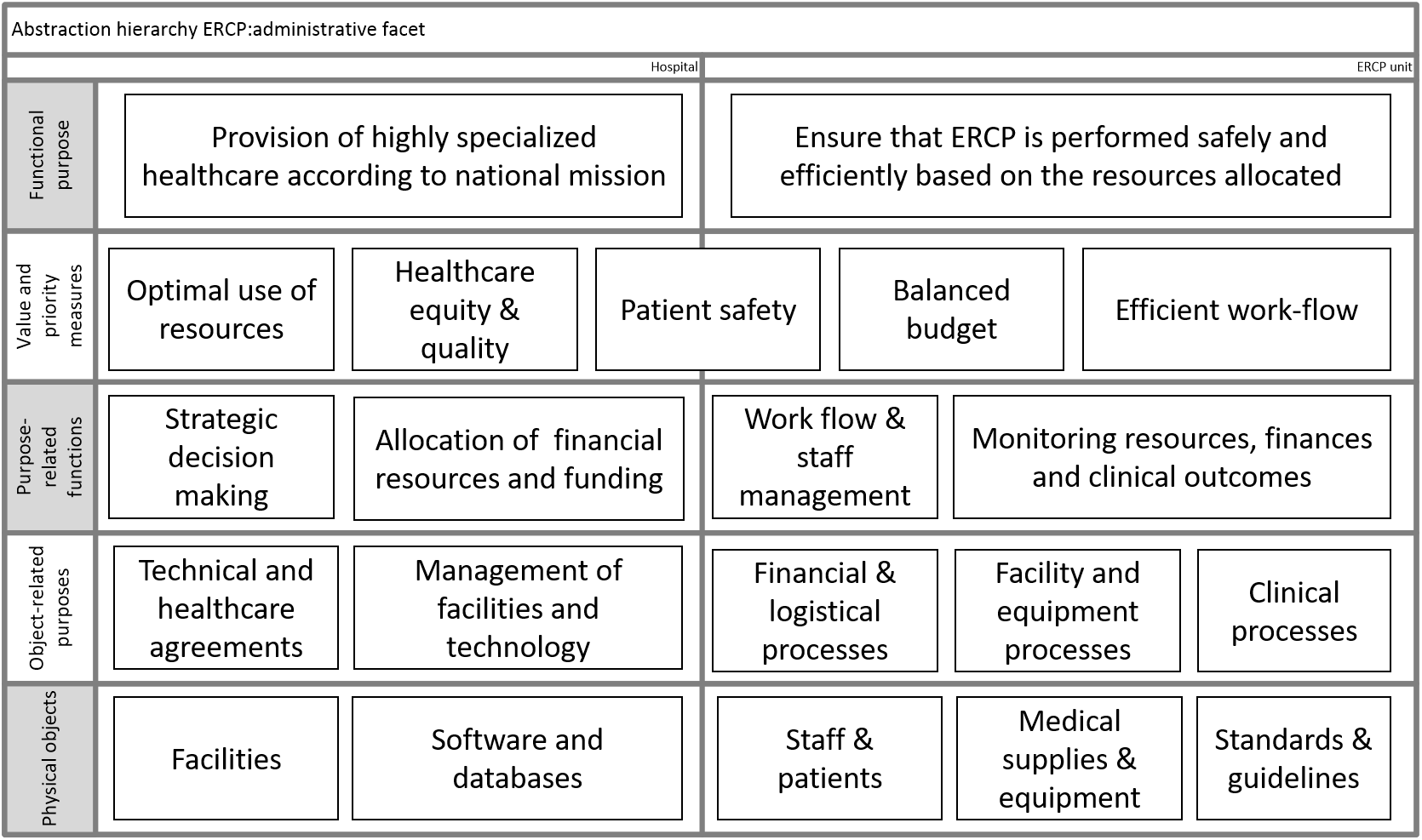

Supplement: Multimedia Appendix 8 [file periop_v4i2e26580_app8.png]
